# An Improved Topology-Potential-Based Community Detection Algorithm for Complex Network

**DOI:** 10.1155/2014/121609

**Published:** 2014-01-29

**Authors:** Zhixiao Wang, Ya Zhao, Zhaotong Chen, Qiang Niu

**Affiliations:** School of Computer Science and Technology, China University of Mining and Technology, Xuzhou, Jiangsu 221116, China

## Abstract

Topology potential theory is a new community detection theory on complex network, which divides a network into communities by spreading outward from each local maximum potential node. At present, almost all topology-potential-based community detection methods ignore node difference and assume that all nodes have the same mass. This hypothesis leads to inaccuracy of topology potential calculation and then decreases the precision of community detection. Inspired by the idea of PageRank algorithm, this paper puts forward a novel mass calculation method for complex network nodes. A node's mass obtained by our method can effectively reflect its importance and influence in complex network. The more important the node is, the bigger its mass is. Simulation experiment results showed that, after taking node mass into consideration, the topology potential of node is more accurate, the distribution of topology potential is more reasonable, and the results of community detection are more precise.

## 1. Introduction

Most complex networks show community structure; that is, groups of vertices that have a higher density of edges within them and a lower density of edges between groups [1]. Identifying community structure is crucial for understanding the structural and functional properties of complex networks [2]. Many works inspired by different paradigms are devoted to the development of community detection [3]. Recently, topology potential theory was introduced to complex network area for community detection [4]. Because of its inherent advantage, such as low time complexity and good performance, this novel theory has attracted plenty of attentions [5–9].

Gan et al. [4] used the topology potential theory to describe the interaction and association among complex network nodes and put forward a community detection algorithm based on topology potential. The community structure can be uncovered by detecting all local high potential areas margined by low potential nodes.

Han et al. [5] proposed an overlapping community detection algorithm based on topology potential. A complex network will be divided into separate communities by spreading outward from each local maximum potential node. The algorithm claims that different nodes play different roles in complex network, such as seed node, overlapping node, and isolated node. Different community roles are identified during spreading process.

Zhang et al. [6] proposed a variable scale network overlapping community identification method based on topology potential. This method defines an identity uncertainty measure to identify overlapping nodes and utilizes the parameter *ξ* to control community scale.

Topology potential calculation is the foundation and key step for the above topology-potential-based community detection methods. In a given network *G* = (*V*, *E*), where *V* = {*v*_*i*_ | *i* = 1,…, *n*} is a set of nodes, *n* is the total number of nodes, *E* = {*v*_*i*_, *v*_*j*_ | *v*_*i*_, *v*_*j*_ ∈ *V*} is a set of edges, and *m* = |*E*| is the total number of edges. The topology potential of any node *v*_*i*_ can be computed as follows:

(1) $$\begin{array}{c} \varphi \left(v_{i}\right)=\sum_{j=1}^{n}\left[m_{j}\times e^{-{\left(d_{ij}/\sigma \right)}^{2}}\right], \end{array}$$

where *φ*(*v*_*i*_) is the topology potential of node *v*_*i*_; *d*_*ij*_ is the distance between node *v*_*i*_ and node *v*_*j*_; *m*_*j*_ is the mass of node *v*_*j*_; and *σ* is impact factor, which is used to control the affecting hops of node. The optimal impact factor can be obtained by using the method described in [4].

In formula (1), the node mass *m*_*j*_ is an important parameter, which will directly affect the value of *φ*(*v*_*i*_). However, almost all the above topology-potential-based community detection methods ignore the difference between nodes and assume *m*_*j*_ = 1. This hypothesis is debatable, and the reasons are described as follows.

On one hand, a node's mass reflects its inherent properties, such as importance and influence. Different nodes have different inherent properties. For example, in social network, the importance of different people is significantly different, and public figures obviously have more influence than general people.

On the other hand, (1) shows that topology potential *φ*(*v*_*i*_) depends on the distance *d* and the mass *m* (the impact factor *σ* is a constant). If we suppose *m* = 1, the calculated topology potential value will deviate from the actual value, and this deviation may affect the precision of community detection.

In order to solve the above problems, this paper puts forward a mass calculation method for complex network nodes, which is inspired from the idea of PageRank [10] algorithm. Node mass calculated by this method can effectively reflect the importance and influence of nodes in complex network. The more important a node is, the bigger its mass is. Simulation experiment results showed that, after taking node mass into consideration, the topology potential of node is more accurate, the distribution of topology potential is more reasonable, and the results of community detection are more precise.

This paper is organized as follows: Section 2 describes the node mass calculation method; Section 3 analyzes the influence of node mass on topology-potential-based community detection; and Section 4 comes to the conclusion of this paper.

## 2. Node Mass Calculation

Apparently, matter particle has its inherent mass. But how to weigh the mass of network nodes? A node's mass should reflect its importance and influence in the complex network. The more important a node is, the bigger its mass should be. Inspired by the idea of PageRank algorithm, this paper puts forward a mass calculation method for complex network nodes.

The PageRank algorithm has been successfully used by Google to evaluate the importance of web pages. Each web page is assigned a PR value to reflect its importance. The algorithm claims that the PR value of a web page can be measured by the number and importance of web pages linking to this page. Generally speaking, the more web pages link to this page, the more important it is. The contributions of these web pages are different: the more important these pages themselves are, the more contribution they make to this page.

Similarly, the importance of a network node can be measured by the number and importance of its neighbor nodes. The more neighbor nodes the node has, the more important it is. The more important its neighbors themselves are, the more important the node is.

**Definition 1**

In a given network *G* = (*V*, *E*), where *V* = {*v*_*i*_ | *i* = 1,…, *n*} is a set of nodes, *n* is the total number of nodes, *E* = {*v*_*i*_, *v*_*j*_ | *v*_*i*_, *v*_*j*_ ∈ *V*} is a set of edges, and *m* = |*E*| is the total number of edges. The mass of any node *v*_*i*_ is defined as follows:

(2) m(vi)=(1−d)+d·(m(v1′)s(v1′)+⋯+m(vi′)s(vi′)+⋯+m(vk′)s(vk′)),

where *m*(*v*_*i*_) is the mass of node *v*_*i*_; *v*_1_′,…, *v*_*i*_′,…, *v*_*k*_′ are neighbors of node *v*_*i*_, *m*(*v*_*i*_′) is the mass of node *v*_*i*_′; 1 ≤ *i* ≤ *k*; *s*(*v*_*i*_′) is the degree of node *v*_*i*_′; and *d* is the damping factor, 0 < *d* < 1.

Definition 1 shows that the value of damping factor *d* will influence the distribution of node mass. The PageRank algorithm set *d* at 0.85 according to a large number of experiments and experiences. Apparently, a suitable damping factor *d* is also needed in node mass calculation.

This paper selected a representative social network—Zachary network to analyze the relationship between damping factor *d* and node mass. The Zachary network is a karate club network with 34 members. This karate club finally split into two communities because of the confliction between its chairman and coach.  Table 1 shows the mass of number 1 node–number 7 node with different damping factors.

As can be seen from Table 1, when *d* is 0, the mass of the seven nodes are all 1, which means that there is no difference in these nodes. With *d* increasing, the mass difference between nodes gradually becomes apparent. When *d* comes to 1, the mass difference reaches the maximum.

**Figure 1:**
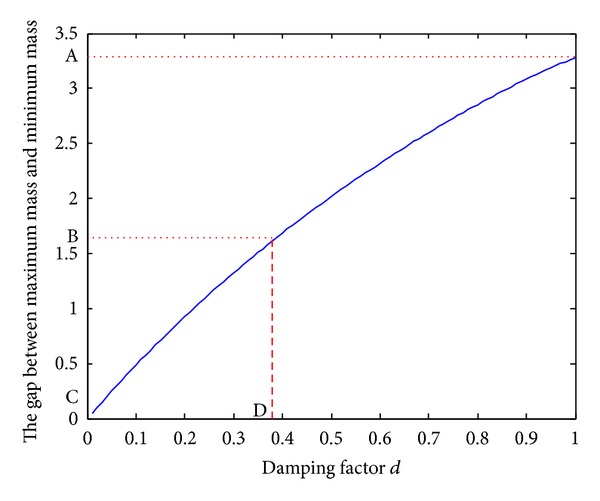
The relationship between damping factor *d* and mass gap.

Figure 1 shows the gap between maximum mass and minimum mass of Zachary network nodes with different damping factors. As can be seen from Figure 1, the gap is increasing with the increasing of *d*, and it almost shows a linear uptrend. In order to ensure mass difference between nodes, highlight important nodes, and meanwhile avoid extreme mass difference, this paper selects *d*, to which the half position (B in Figure 1) between no mass difference (C in Figure 1) and the biggest mass difference (A in Figure 1) corresponds, as optimal value. For the Zachary network, the corresponding optimal damping factor is 0.38 (D in Figure 1).

Node mass calculated by our method can effectively reflect the importance and influence of nodes in complex network. The more important a node is, the bigger its mass is. After taking node mass into consideration, the topology potential of node will be more accurate, and the distribution of topology potential will be more reasonable. Now that mass *m* reflects the influence of node in whole complex network, thus, the topology potential, which depends on the distance *d* and the mass *m*, is of global characteristic to some extent. This global characteristic will be meaningful for community detection.

## 3. Simulation Experiments

This section will empirically analyze the influence of node mass on three typical topology-potential-based community detection methods. These three methods come from literature [4], literature [5], and literature [6]. In this paper, they are called Gan, Han, and Zhang, respectively.

Simulation program was implemented using scientific computing software MATLAB in the Windows environments. The experiment data include two complex networks: one is a real world network—Dolphin social network, which comes from http://www-personal.umich.edu/~mejn/netdata/; and the other is an artificial network, which is generated by LFR-Benchmark generator [11]. LFR-Benchmark is a network generator, which produces networks with power-law degree distribution and with implanted communities within the network.

For each network, there are two schemes: one is “without mass” scheme, which ignores node difference and sets *m* = 1, and the other is “with mass” scheme, which takes node mass into consideration; node mass is computed according to Definition 1. We analyzed the topology potential of nodes and community detection results with these two schemes.

### 3.1. Artificial Complex Network

**Figure 2:**
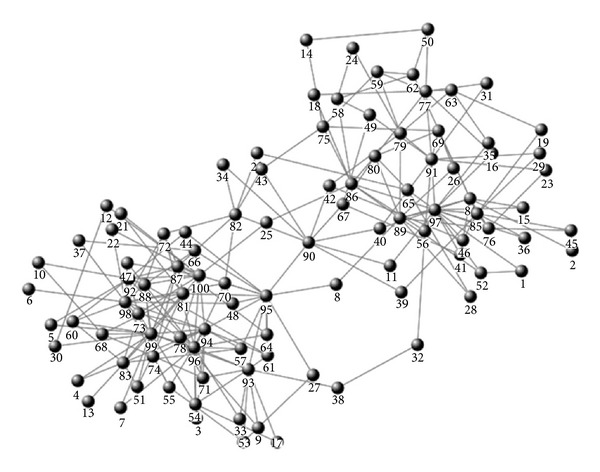
Artificial complex network.

The artificial complex network is generated by the LFR-Benchmark generator. The node number is 100, the edge number is 230, the average degree is 4.6, and the implanted community number is 2. The structure of the artificial complex network is shown in Figure 2.

#### 3.1.1. The Influences of Node Mass on Topology Potential

Table 2 shows the topology potential of number 1 node–number 20 node with two schemes. As seen from Table 2, the topology potential of artificial network nodes shows obvious changes after taking node mass into consideration.

Table 3 shows the top 20 nodes with the biggest topology potential in two schemes. As seen from Table 3, the top 20 nodes sequence changes from the fourth biggest node after taking node mass into consideration. The change of node sequence implies the change of topology potential distribution, which may affect community detection results.

#### 3.1.2. The Influences of Node Mass on Community Detection Results

The artificial complex network contains two communities: the community *C*_97_ and the community *C*_99_. The representative node of *C*_97_ is number 97 node, and the representative node of *C*_99_ is number 99 node.

(*1) The Gan Method.* The Gan method first identifies internal nodes and boundary nodes and then uses defined benefit function to determine which community a boundary node belongs to. For the “without mass” scheme, the boundary nodes identified by the Gan method are {8, 11, 20, 25, 32, 39, 40, 41, 42, 43, 49, 67}, with a total number of 12. For the “with mass” scheme, the boundary nodes identified by the Gan method are {8, 11, 20, 25, 32, 39, 40, 42, 43, 49, 67}, with a total number of 11. Obviously, after taking node mass into consideration, the boundary nodes reduced from 12 to 11; thereby it can lighten the load of determining which community a boundary node belongs to. As can be seen from Figure 2, number 41 node is apparently the internal node of community *C*_97_. But if we do not take node mass into consideration, this node is regarded as boundary node by mistake.

(*2) The Zhang Method.* Zhang method uses the same strategy as the Gan method to identify internal nodes and boundary nodes. The only difference is the way of determining which community a boundary node belongs to. Therefore, For the “without mass” scheme, the boundary nodes identified by the Zhang method are also {8, 11, 20, 25, 32, 39, 40, 41, 42, 43, 49, 67}, with a total number of 12. When we take node mass into consideration, the boundary nodes identified by the Zhang method are also {8, 11, 20, 25, 32, 39, 40, 42, 43, 49, 67}, with a total number of 11.

*(3) The Han Method.* The community detection results remain the same with these two schemes. The reason is as follows: the Han method simply utilizes topology potential to find local maximum topology potential nodes, that is, representative nodes of communities, and then it uses a strategy similar to modularity to determine which community nodes it belongs to. Whether we take node mass into consideration or not, local maximum topology potential nodes are not changed (always number 97 node and number 99 node), and complex network structure is steadiness; therefore, community detection results remain the same.

### 3.2. Dolphin Social Network

**Figure 3:**
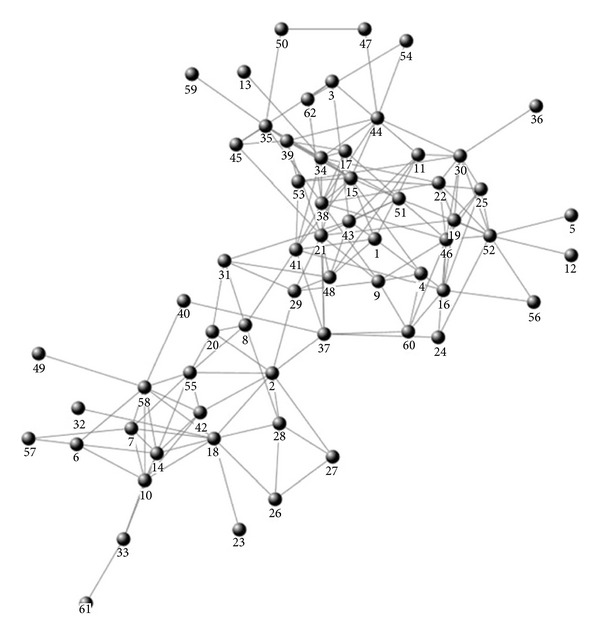
Dolphin social network.

The Dolphin social network describes the frequent associations between 62 dolphins in a community living off Doubtful Sound, New Zealand. The structure of the Dolphin social network is shown in Figure 3.

#### 3.2.1. The Influences of Node Mass on Topology Potential

Table 4 shows the topology potential of number 1 node–number 20 node with two schemes. As seen from Table 4, the topology potential of Dolphin nodes shows obvious changes after taking node mass into consideration.

Table 5 shows the top 20 nodes with the biggest topology potential in two schemes. As seen from Table 5, the top 20 nodes sequence changes from the fifth biggest node after taking node mass into consideration. This change may affect community detection results.

#### 3.2.2. The Influences of Node Mass on Community Detection Results

Dolphin social network contains two communities: the community *C*_15_ and the community *C*_18_. The representative node of *C*_15_ is number 15 node, and the representative node of *C*_18_ is number 18 node.

(*1) The Gan Method.* For the “without mass” scheme, the boundary nodes identified by the Gan method are {2, 7, 8, 20, 26, 27, 28, 29, 31, 40, 42, 55, 57}, with a total number of 13. For the “with mass” scheme, the boundary nodes identified by the Gan method are {4, 8, 20, 24, 29, 31, 37, 40, 60}, with a total number of 9. Obviously, after taking node mass into consideration, the boundary nodes reduced from 13 to 9; thereby it can lighten the load of determining which community a boundary node belongs to.

As can be seen from Figure 3, number 7 node, number 26 node, number 27 node, number 42 node, and number 57 node are apparently the internal nodes of community *C*_18_. But if we do not take node mass into consideration, these nodes are regarded as boundary nodes by mistake.

There emerge some new boundary nodes for the “with mass” scheme, such as number 24 node and number 60 node. In Figure 3, these two nodes locate in the overlapping area of community *C*_18_ and community *C*_15_, and they all directly connect to number 37 node, which definitely is an overlapping node. So it is reasonable to claim that number 24 node and number 60 node are boundary nodes.

(*2) The Zhang Method.* Whether for the “without mass” scheme or the “with mass” scheme, the boundary nodes identified by Zhang method are all the same as Gan method, and after taking node mass into consideration, the boundary nodes reduced from 13 to 9. The reason is the same as that explained in Section 3.1.

*(3) The Han Method.* The community detection results remain the same with these two schemes. The reason is the same as that explained in Section 3.1.

## 4. Conclusions

Topology potential theory is a new community detection theory for complex network. At present, almost all topology-potential-based community detection methods assume that network nodes have the same mass. This hypothesis leads to inaccuracy of topology potential calculation and then decreases the precision of community detection. Inspired by the idea of PageRank algorithm, this paper puts forward a novel mass calculation method for complex network node. A node's mass obtained by our method can effectively reflect its importance and influence in complex network. The more important a node is, the bigger its mass is. Simulation experiment results showed that, after taking node mass into consideration, the topology potential of node is more accurate, the distribution of topology potential is more reasonable, and the results of community detection are more precise.

## Figures and Tables

**Table 1:** The relationship between damping factor *d* and node mass.

Damping factor *d*	Number 1 node	Number 2 node	Number 3 node	Number 4 node	Number 5 node	Number 6 node	Number 7 node
0.00	1.000000	1.000000	1.000000	1.000000	1.000000	1.000000	1.000000
0.10	1.395255	1.126644	1.112363	1.021542	0.966275	1.013809	1.013809
0.20	1.747079	1.237061	1.219573	1.041998	0.935492	1.025750	1.02575
0.30	2.061667	1.335101	1.323713	1.062184	0.907004	1.035298	1.035298
0.40	2.343918	1.424051	1.426751	1.082954	0.880128	1.041800	1.041800
0.50	2.597738	1.506913	1.530693	1.105297	0.854066	1.044337	1.044337
0.60	2.826218	1.586712	1.637788	1.130467	0.827759	1.041493	1.041493
0.70	3.031637	1.666869	1.750830	1.160193	0.799622	1.030909	1.030909
0.80	3.215021	1.751764	1.873724	1.197029	0.766916	1.008239	1.008239
0.90	3.374151	1.847568	2.01265	1.244893	0.724111	0.964480	0.964480
1.00	3.495605	1.963056	2.178759	1.309709	0.658029	0.878151	0.878151

**Table 2:** The topology potential of artificial network nodes with two schemes.

Node	Without mass	With mass	Node	Without mass	With mass
Node 1	2.4140	1.8383	Node 11	2.5175	2.0233
Node 2	2.1035	1.4705	Node 12	2.2415	1.6444
Node 3	2.5520	1.7553	Node 13	2.5520	1.3730
Node 4	2.5175	1.8146	Node 14	2.5865	1.4425
Node 5	2.5175	1.8885	Node 15	2.4140	1.9363
Node 6	1.9140	1.2719	Node 16	2.2415	1.9283
Node 7	2.6900	1.9954	Node 17	2.4140	1.5655
Node 8	2.6210	1.7898	Node 18	3.3280	1.5591
Node 9	2.2760	1.5757	Node 19	3.0175	1.6472
Node 10	2.0000	1.4835	Node 20	2.8105	1.4862

**Table 3:** The top 20 nodes of the artificial complex network.

Serial number	Without mass	With mass	Serial number	Without mass	With mass
1	Node 99	Node 99	11	Node 91	Node 91
2	Node 97	Node 97	12	Node 90	Node 88
3	Node 100	Node 100	13	Node 85	Node 85
4	Node 98	Node 94	14	Node 87	Node 87
5	Node 95	Node 98	15	Node 88	Node 86
6	Node 94	Node 96	16	Node 86	Node 90
7	Node 96	Node 95	17	Node 81	Node 84
8	Node 93	Node 93	18	Node 82	Node 76
9	Node 92	Node 89	19	Node 84	Node 81
10	Node 89	Node 92	20	Node 78	Node 78

**Table 4:** The topology potential of Dolphin nodes with two schemes.

Node	Without mass	With mass	Node	Without mass	With mass
Node 1	6.2895	4.5944	Node 11	5.3610	3.9025
Node 2	7.0212	5.4907	Node 12	2.3973	1.7321
Node 3	4.1513	2.9848	Node 13	2.3973	1.6296
Node 4	4.1605	2.8547	Node 14	6.4585	5.5909
Node 5	2.3973	1.7321	Node 15	9.6098	7.6395
Node 6	3.8699	3.2161	Node 16	6.4678	4.7977
Node 7	5.4456	4.6391	Node 17	6.1957	4.8912
Node 8	5.4548	3.8740	Node 18	7.1057	6.1104
Node 9	6.2895	4.6118	Node 19	6.9367	5.4700
Node 10	5.8114	5.0576	Node 20	4.3388	3.1913

**Table 5:** The top 20 nodes of Dolphin social network.

Serial number	Without mass	With mass	Serial number	Without mass	With mass
1	Node 15	Node 15	11	Node 39	Node 51
2	Node 38	Node 38	12	Node 18	Node 39
3	Node 46	Node 46	13	Node 2	Node 14
4	Node 34	Node 34	14	Node 19	Node 44
5	Node 21	Node 52	15	Node 44	Node 2
6	Node 41	Node 30	16	Node 58	Node 19
7	Node 30	Node 18	17	Node 16	Node 37
8	Node 52	Node 21	18	Node 14	Node 22
9	Node 37	Node 41	19	Node 22	Node 10
10	Node 51	Node 58	20	Node 9	Node 25
